# Characterization of Al_2_O_3_ Matrix Composites Fabricated via the Slip Casting Method Using NiAl-Al_2_O_3_ Composite Powder

**DOI:** 10.3390/ma15082920

**Published:** 2022-04-16

**Authors:** Justyna Zygmuntowicz, Katarzyna Konopka, Marek Krasnowski, Paulina Piotrkiewicz, Jan Bolek, Marcin Wachowski, Radosław Żurowski, Mikołaj Szafran

**Affiliations:** 1 Faculty of Materials Science and Engineering, Warsaw University of Technology, 141 Woloska St., 02-507 Warsaw, Poland; katarzyna.konopka@pw.edu.pl (K.K.); marek.krasnowski@pw.edu.pl (M.K.); paulina.piotrkiewicz.dokt@pw.edu.pl (P.P.); jan.bolek2.stud@pw.edu.pl (J.B.); 2 Faculty of Mechanical Engineering, Military University of Technology, 2 Gen. S. Kaliskiego St., 00-908 Warsaw, Poland; marcin.wachowski@wat.edu.pl; 3 Faculty of Chemistry, Warsaw University of Technology, 3 Noakowskiego St., 00-664 Warsaw, Poland; rzurowski@ch.pw.edu.pl (R.Ż.); mikolaj.szafran@pw.edu.pl (M.S.)

**Keywords:** NiAl-Al_2_O_3_, composite, slip casting, Al_2_O_3_ matrix

## Abstract

This work aimed to characterize Al_2_O_3_ matrix composites fabricated by the slip casting method using NiAl-Al_2_O_3_ composite powder as the initial powder. The composite powder, consisting of NiAl + 30 wt.% Al_2_O_3_, was obtained by mechanical alloying of Al_2_O_3_, Al, and Ni powders. The composite powder was added to the Al_2_O_3_ powder to prepare the final powder for the slip casting method. The stained composite samples presented high density. EDX and XRD analyses showed that the sintering process of the samples in an air atmosphere caused the formation of the NiAl_2_O_4_ spinel phase. Finally, the phase composition of the composites changed from the initial phases of Al_2_O_3_ and NiAl to Al_2_O_3_, Ni, and NiAl_2_O_4_. However, in the area of Ni, fine Al_2_O_3_ particles remaining from the initial composite powder were visible. It can be concluded that after slip casting, after starting with Al_2_O_3_ and the composite powder (NiAl-Al_2_O_3_) and upon sintering in air, ceramic matrix composites with Ni and NiAl_2_O_4_ phases, complex structures, high-quality sintered samples, and favorable mechanical properties were obtained.

## 1. Introduction

Ceramic matrix composites (CMCs) are being developed because of the potential for the extensive application of these materials. By combining ceramics with metals or an intermetallic phase and shaping new microstructures, CMCs have a broad spectrum of mechanical and functional properties. Data in the literature show that CMCs are engineering materials used for many industrial applications such as cutting tools, wear resistance components, resistance resistors, and biomaterials [1,2,3,4,5].

The mechanical and functional properties depend on the microstructures of the composites. Various options, starting with selecting the ceramic–metal or ceramic–intermetallic system, using micro- and nanoparticles as reinforcement inclusions, and the distribution of the incorporation phases are the key factors responsible for the microstructures and final properties of CMCs [1,2,3]. In recent years, composites with complex structures have emerged. Such composites have improved fracture toughness and other properties [4,5,6,7].

The paper presents ceramic matrix composites with complex microstructures, the method of their fabrication, and the characterization of their microstructures and properties. Mainly, complex microstructures can be obtained by using an earlier prepared composite powder. We proposed using an intermetallic–ceramic composite powder (NiAl-Al_2_O_3_) obtained by mechanical alloying as an initial powder mixed with Al_2_O_3_ powder. After powder consolidation, the Al_2_O_3_-(NiAl-Al_2_O_3_) composite was expected.

The NiAl-Al_2_O_3_ system is known in the literature. High melting temperature, low density, and corrosion resistance [8] are the main important features of nickel aluminide. These properties are important for high-temperature and chemical-resistant applications. However, NiAl has low toughness and ductility at room temperature [8], limiting this phase’s application. Because of this, NiAl is used as a matrix for composites with the addition of reinforcements that improve these negative features, e.g., NiAl-WC [9], NiAl-TiC-Al_2_O_3_ [10], NiAl-B [11], and NiAl-Al_2_O_3_ [12,13]. In this sense of improving the negative properties of NiAl, which was also present in this paper’s composite powder, NiAl-Al_2_O_3_ was used to prepare the final bulk composite, Al_2_O_3_-(NiAl-Al_2_O_3_). However, composites of Al_2_O_3_-NiAl, but fabricated from Al_2_O_3_ and NiAl, are also described in the literature [14,15,16]. Improvement of the fracture toughness of these materials is reported in [14,15,16]. In our concept, using the composite powder, NiAl-Al_2_O_3_, mixed with Al_2_O_3_ and then consolidated provides the advantage of obtaining composites with microstructures that consist of a ceramic matrix and two-phase reinforcement—NiAl inside of which are distributed fine Al_2_O_3_ particles. Finally, composites with complex structures can be fabricated. In such composites, the fracture toughness can effectively be enhanced. Simultaneously, the properties of NiAl together with those of Al_2_O_3_, especially high melting points and chemical resistance, can dedicate the composite, Al_2_O_3_-(NiAl-Al_2_O_3_), to high-temperature applications.

The results of our own previously prepared works were concentrated on the preparation and characterization of NiAl-Al_2_O_3_ composite powder. The composite powder was prepared by mechanical alloying from powder mixtures containing Ni-50 at.% and Al with a contribution of 10 wt.% or 20 wt.% of Al_2_O_3_ [17]. However, positive attempts to use more than—30 wt.% Al_2_O_3_ were also made. This is important, because in the literature, an improvement in the properties of NiAl by adding Al_2_O_3_ was observed in the range 13 to 55 vol.% in ceramics [12,18], which meant that our choice of Al_2_O_3_ addition was within this range.

The results revealed that the composite powder could be used to reinforce ceramics and to fabricate CMCs with complex microstructures [17]. In such composites, new phases, such as spinel NiAl_2_O_4_, can also be formed during air sintering. Because of this, it was also considered in our experiments.

The slip casting method was selected for the composites’ fabrication. The main reason for selecting this method was that slip casting provides high control over the molding process and allows for obtaining samples with complicated shapes without the green machining step (near-net shaping). It also guarantees the good quality and homogeneity of the casted samples, which is crucial in the fabrication of ceramics and ceramic matrix composites. Moreover, colloidal processing, as a slip casting method, is essential for producing dense materials. It is possible to form complex shapes. An important advantage is also the possibility of producing ceramic matrix composites with the addition of a metal or intermetallic phase [19].

In the paper, the process of preparing the NiAl + 30 wt.% Al_2_O_3_ composite powder, the fabrication of the Al_2_O_3_-(NiAl-Al_2_O_3_) composite by slip casting, and the microstructures and properties are presented. Scanning electron microscopy observations of the microstructures and fractures were made with quantitative analysis. The results in terms of density, hardness, and K_IC_ are discussed.

## 2. Experimental

### 2.1. Materials

The α-Al_2_O_3_ powder of alumina with the symbol, TM-DAR (Taimei Chemicals Co., Ltd., Tokyo, Japan), and an average particle size of 0.1 μm, density of 3.9 g/cm^3^, and 99.99% purity was used as ceramic powder. One of the metal powders used was aluminum (ABCR GmbH & Co.KG, Karlsruhe, Germany) with a nominal particle size of 44 µm, theoretical density of 2.7 g/cm^3^, and purity of 99.7%. The second metal powder was nickel powder (ABCR GmbH & Co.KG, Karlsruhe, Germany) with an average particle size equal to 3–7 µm and a density of 8.9 g/cm^3^. As dispersing agents for the slurries, diammonium hydrocitrate (DAC) (Sigma–Aldrich, St. Louis, MO, USA) and citric acid (CA) (Sigma–Aldrich) were used. Deionized water was used as a solvent.

### 2.2. Preparation of the Samples

The first step in the investigation was mechanical alloying to prepare a composite powder containing NiAl intermetallic and 30 wt.% Al_2_O_3_ (called compo-powder). A SPEX 8000D high-energy shaker ball mill (SPEX^®^ SamplePrep, Metuchen, NJ, USA) was used for milling of a blend consisting of Ni50Al50 (at.%) elemental powders with the addition of 30 wt.% Al_2_O_3_ powder. An 8:1 ball-to-powder weight ratio was used. The milling processes were carried out under an argon atmosphere. Balls with diameters of 10 and 12 mm were used.

The composites were produced by the slip casting method. The principle of slip casting is not a straightforward filtration method but a controlled destabilization of a ceramic or ceramic–metal dispersion [20]. Stable ceramic–metal or ceramic slurries are poured into a gypsum substrate on which are placed polypropylene molds or into a dry porous mold of plaster. In this technique, the porous gypsum molds remove a part of the solvent contained in the suspension.

Consequently, the solid phase content of the suspension in contact with the interface increases, and a ceramic–metal or ceramic body is deposited [20,21]. After the solvent is removed, the spacing decreases. The distance between particles is less than the distance from the energy barrier, and the attractive forces become predominant [20,21]. This leads to a relatively dense and homogeneous packing of particles similar to that in pressed samples.

Within the framework of the present work, three series of samples were made: Series I—100 vol.% of Al_2_O_3_; Series II—Al_2_O_3_ + 2.5 vol.% of compo-powder (NiAl + 30 wt.% Al_2_O_3_); Series III—Al_2_O_3_ + 5 vol.% of compo-powder (NiAl + 30 wt.% Al_2_O_3_). Detailed compositions of each suspension are listed in Table 1. The manufacturing process took place in several stages. Two dispersing agents, DAC and CA, were dissolved in deionized water. Then, the appropriate amounts of ceramic (Al_2_O_3_) and compo-powder (NiAl + 30% by weight of A_2_O_3_) were dispersed. The suspension was mixed using a PM400 planetary ball mill (Retsch, Haan, Germany) for 60 min at 300 rpm. In the investigation, the suspensions were mixed in an alumina jar. Six Al_2_O_3_-made ceramic balls were used as the grounding medium. Next, the suspension was mixed and degassed on a THINKY ARE-250 device (Thinky Corporation, Tokyo, Japan). The slurry was cast on a gypsum substrate on which polypropylene (PP) molds 20 mm in diameter and 5 mm in height were placed. After the suspension was poured into the molds, the solvent was drained by capillary forces by the open porosity of the gypsum mold. In the next step, the green bodies were dried for 48 h at 35 °C in a laboratory drier. After being dried, the material was removed from the PP molds and green machined to remove the eventual roughness. The samples were sintered in a Carbolite STF 16/75/450 furnace: heating at 2 °C/min up to 1450 °C; holding for 2 h at 1450 °C; cooling at 5 °C/min. Air was used as the sintering atmosphere.

### 2.3. Research Procedures

The actual density of the NiAl + 30 wt.% Al_2_O_3_ powder was measured by a helium pycnometer (AccuPyc 1340 II, Micromeritics, Norcross, GA, USA). Density measurements were estimated using the ASTM D3766 standard [22].

The relative density, soaking, and open porosity of the samples fabricated by slip casting were characterized by the Archimedes method. Measurements were carried out according to the European Standard EN623-2 [23]. Density measurements were made using ten specimens for each type of material examined.

X-ray diffractometry (XRD) analyses of powder after MA and the samples were carried out on a Rigaku MiniFlex II diffractometer (Rigaku Corporation, Tokyo, Japan) using CuKα radiation (λ = 1.54178 Å) working at 30 kV and 15 mA in a step-scanning mode with a step size of 0.05° and counting time of 3 s at diffraction angles 2θ ranging from 23° to 120°.

The Williamson–Hall [24] method was used to estimate the mean crystallite size of the final product of the NiAl phase in the mechanical alloying. The instrumental broadening was subtracted from the experimental breadth to obtain the physical broadening of each diffraction line.

The microstructure of the powder and sintered samples was studied by scanning electron microscopy (JEOL JSM-6610 SEM, Tokyo, Japan). The microscope had backscattered electron (BSE) and secondary electron (SE) detectors. A voltage of 15 kV was used throughout the observations. Surface microanalysis was conducted using an X-Max-type energy-dispersive X-ray spectrometer (EDS, Oxford, UK) to determine the elemental concentration in the powder obtained after mechanical alloying and the samples obtained by the slip casting method.

**Figure 1:**
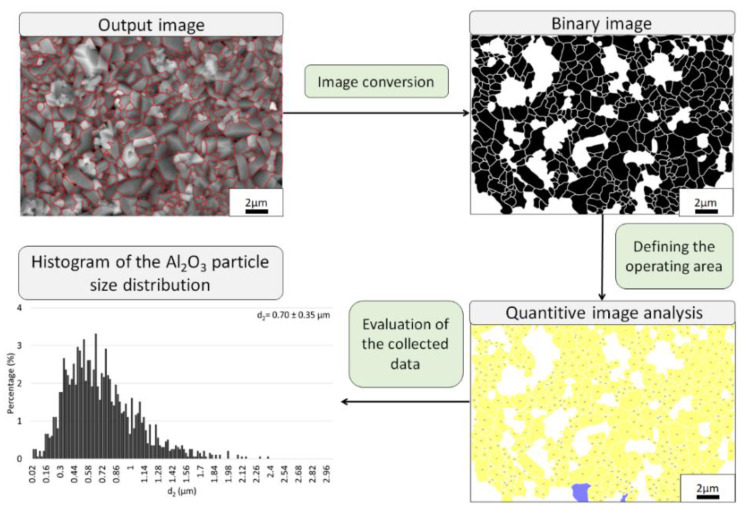
Schematic of the image processing methodology used to obtain the Al_2_O_3_ percentage share of the particle size distribution in the samples.

A quantitative microstructural characterization was performed with a MicroMeter v.086b computer image analyzer. Raw Al_2_O_3_ powder and Al_2_O_3_ grains in the composites were examined [25,26]. Quantitative characterization of the microstructure of the samples was carried out on microphotography of randomly determined areas of the fracture of the samples. This procedure provides the ability to obtain details of the actual size of Al_2_O_3_ in the sample. The schematic of the image processing methodology to obtain the percentage share of the particle size distribution of Al_2_O_3_ powder in the samples is shown in Figure 1. Subsequently, the following shape parameters were verified: elongation (α = d_max_/d_2_), (W = p/p_c_), and curvature of the grain boundary (R = p/(π·d_2_)). The formulas used were: d_2_—diameter of a circle of the same surface as the surface of the examined grain [μm]; d_max_—maximum diameter of grain projection [μm]; p—perimeter of grain [μm]; p_c_—Cauchy perimeter [μm] [25,26].

The Vickers technique measured the hardness of the samples. Hardness was estimated on the polished sample surface. A 10 s holding time was used under a load of 10 kg during the measurements. An HVS-30T hardness tester (Huatec Group Corporation, Beijing, China) was used in the investigation. At least ten measurements were performed for individual samples. The corresponding indentation sizes were determined using diagonals and calculated using a Nikon Eclipse LV15ON light microscope (Nikon, Tokyo, Japan).

The fracture toughness investigations were carried out using the indentation method. This method is based on the measurement of the cracks that develop from the corners of the indentation formed due to the hardness measurement using the Vickers method and, subsequently, calculating the value of the K_IC_ coefficient. The formula proposed by Anstis was used in this study [27]:

$$K_{\mathrm{IC}}=0.016{\left(\frac{E}{\mathrm{HV}}\right)}^{0.5}\cdot \frac{F}{c^{1.5}}$$

where E—Young’s modulus; HV—Vickers hardness; F—total load applied; c—central crack length. 

## 3. Results and Discussion

### 3.1. Description of the Raw Powders

**Figure 2:**
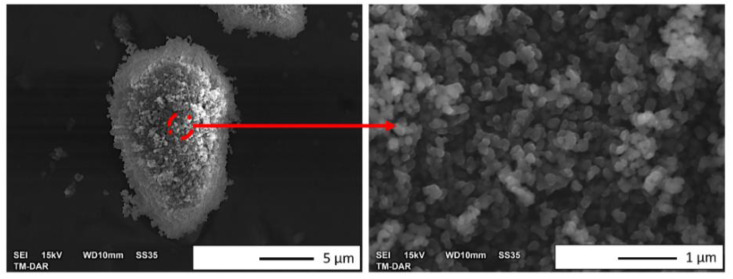
SEM images of the morphology of the Al_2_O_3_ powder.

**Figure 3:**
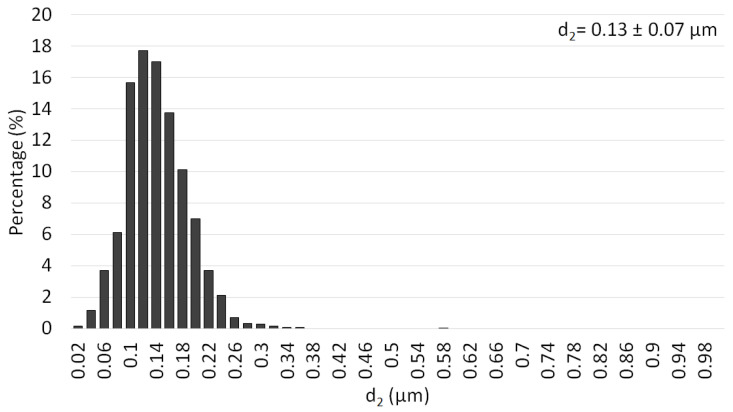
Histogram of the particle size distribution of the Al_2_O_3_ powder.

The morphology of Al_2_O_3_ is presented in Figure 2, while Figure 3 shows the Al_2_O_3_ particle size distribution histogram. It has been stated that the Al_2_O_3_ powder used tends to form agglomerates. Based on observations, it can also be noticed that the shapes of the Al_2_O_3_ particles were slightly heterogeneous. The analysis showed that the Al_2_O_3_ has a particle size equal to 0.13 ± 0.07 µm. Based on the histogram, it was found that the actual size of the powders was close to the manufacturer’s stated size. Similar conclusions were obtained in previous work [17,28].

**Figure 4:**
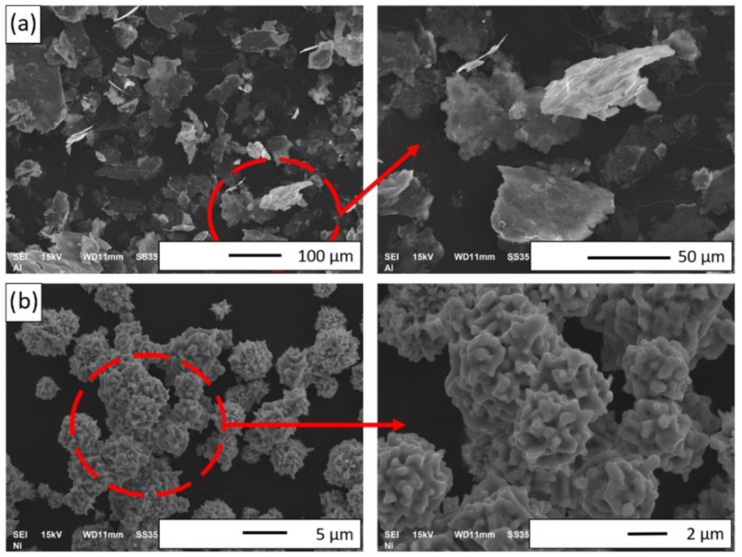
SEM images of initial powders: (**a**) Al and (**b**) Ni.

The SEM images of the metal powders are shown in Figure 4. The micrographs obtained reveal that the Al powder was characterized by a flaky morphology (Figure 4a). Most of the Al flakes were not crushed and thick. The nickel particles demonstrated a cubic shape (Figure 4b). Furthermore, SEM microphoto images of the Ni powder exhibited numerous protrusions present on the surface of its particles.

The densities of the Al_2_O_3_, Ni, and Al powders were measured with a helium pycnometer equal to 3.9828, 8.957, and 3.402 g/cm^3^, respectively. The obtained values of the densities were close to those given by the manufacturers. 

### 3.2. Description of NiAl + 30 wt.% Al_2_O_3_ Composite Powder

**Figure 5:**
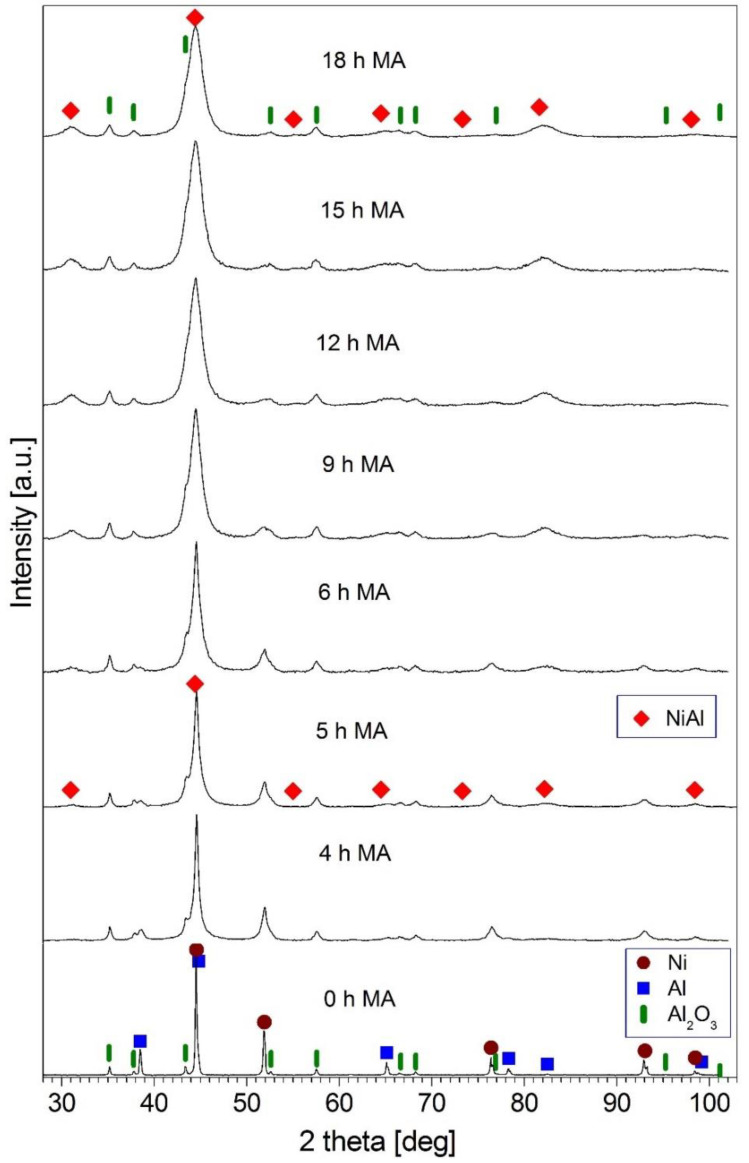
XRD patterns of the (Ni-50 at.% Al) + 30 wt.% Al_2_O_3_ powder blend mechanically alloyed for the times quoted.

Phase changes in the (Ni-50 at.% Al) + 30 wt.% Al_2_O_3_ powder mixture during mechanical alloying were monitored based on XRD examination. The XRD patterns of the (Ni-50 at.% Al) + 30 wt.% Al_2_O_3_ sample after various milling times are shown in Figure 5. In the XRD pattern taken after 5 h of mechanical alloying, peaks of a NiAl intermetallic phase (ICDD 20-0019) appeared. Simultaneously, the intensity of the Ni and Al peaks decreased. The intensity of these peaks decreased gradually with an increasing milling time and, finally, they vanished. The observed formation of a NiAl phase and the disappearance of the Al and Ni were analogous to that described earlier for the mechanical alloying of the Ni-50 at.% Al powder mixture [29] and for the (Ni-50 at.% Al) + X wt.% Al_2_O_3_ (X = 10, 20) powder mixtures [17,30] carried out using the same ball mill as in the present work. In the patterns presented in Figure 5, the diffraction peaks of Al_2_O_3_ (ICDD 10-0137) were unchanged for all milling times. The phase composition of the milling product (after 18 h of milling) was the NiAl (ICDD 20-0019) intermetallic phase (at least partially ordered) and Al_2_O_3_. For the (Ni-50 at.% Al) + X wt.% Al_2_O_3_ (X = 10, 20) powder mixtures [17,30], where a similar phase evolution was observed, phase changes occurred faster than for the mixture containing 30 wt.% of Al. The mechanical alloying process required 12 and 15 h of milling for the mixture containing 10 and 20 wt.% of Al, respectively [17]. The impact of the amount of reinforcing phase on the phase transformation rate and the NiAl phase formation during mechanical alloying is reported in [11]. In the case of the mechanical alloying of Ni-Al-B powder mixtures, it was found that with the increase in the amount of boron in the mixture, the formation of the NiAl phase and disappearance of the Al and Ni required longer milling time [11]. The influence of the amount of reinforcing phase on the phase transformation during mechanical alloying was observed for the Fe-Al-B powder mixtures [31].

**Figure 6:**
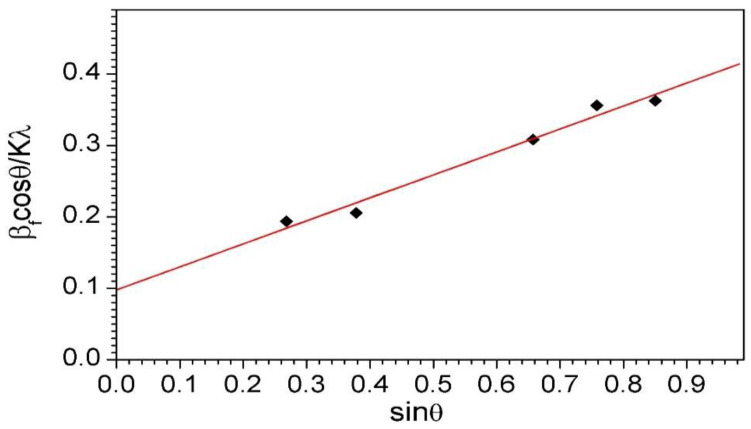
Williamson–Hall plot for the NiAl phase in the final milling product.

The mean crystallite size of the NiAl phase in the final milling product, estimated using the Williamson–Hall method, was 10 nm [24]. Figure 6 presents the corresponding Williamson–Hall plot for the NiAl intermetallic phase.

**Figure 7:**
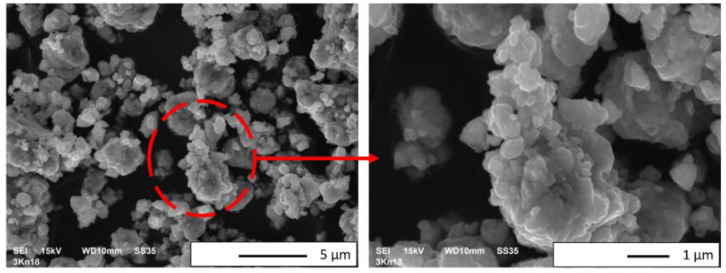
SEM micrographs of the NiAl + 30 wt.% Al_2_O_3_ powder after mechanical alloying for 18 h.

The microstructures of NiAl + 30 wt.% Al_2_O_3_ powder particles after 18 h of mechanical alloying are shown in the SEM micrographs in Figure 7. It is noted that the obtained powder consisted of agglomerates of submicron particles that formed. The single particles of the NiAl + 30 wt.% Al_2_O_3_ powder were almost spherical in shape. Furthermore, it was found that the sizes of the NiAl + 30 wt.% Al_2_O_3_ powder ranged from below 0.5 up to 10 µm. The density, measured using a helium pycnometer, of the NiAl + 30 wt.% Al_2_O_3_ powder was 4.901 g/cm^3^.

**Figure 8:**
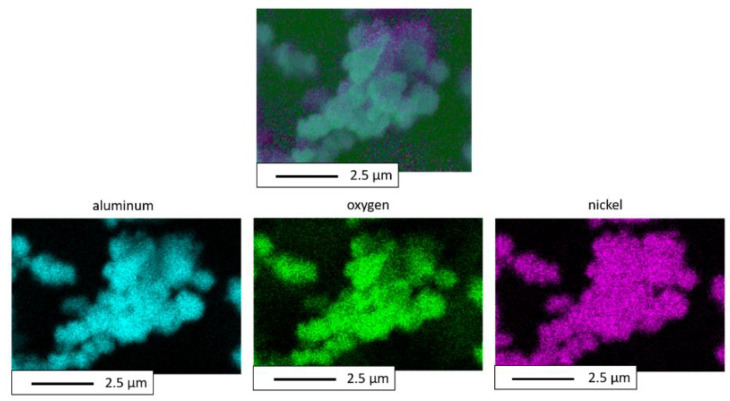
EDS mapping of the NiAl + 30 wt.% Al_2_O_3_ powder after mechanical alloying for 18 h.

The distribution of elements measured by EDS microanalysis revealed that the NiAl + 30 wt.% Al_2_O_3_ powder was formed of Al, Ni, and O. A map of the distribution of the elements for the powder after mechanical synthesis is presented in Figure 8.

### 3.3. Characterization of the Samples Obtained by Slip Casting

The physical properties of the specimens obtained are presented in Table 2. The formation of the samples by slip casting gave a relative density value for all series at a level of 99%. The high densification of the samples produced was probably influenced by the following factors: particle size, particle size distribution, powder morphology, and well-homogenized slurry. These parameters significantly affected the slip-cast body’s packing density and the sintered sample’s microstructure. To prepare samples with uniform microstructures, the starting materials were spherical powders with a tightly controlled powder size distribution, and the small sizes were perfect. For the fabrication of samples, powders with an almost spherical shape were used. The experiment used Al_2_O_3_ powders with particles equal to 0.13 ± 0.07 µm and compo-powder after mechanical synthesis powder with particle sizes equal to 0.5 up to 10 µm. The open porosity and soaking of all of the sintered samples were very low, and the former was close to zero.

Furthermore, the samples consisting of only Al_2_O_3_ had a linear shrinkage of 14.23 ± 0.04%, while Series II had a linear shrinkage of 14.77 ± 0.57%. In comparison, Series III was characterized by a linear shrinkage equal to 15.42 ± 0.75% (measured at the height of the sintered sample). Based on the obtained shrinkage results, it was found that the addition of the metallic phase slightly increased the linear shrinkage. Moreover, it was found that the addition of the composite powder caused a slight decrease in the volume of the shrinkage. The lowest shrinkage was characterized by composites containing 5 vol.% of (NiAl-Al_2_O_3_) at 34%. This can be connected to the higher contribution of the NiAl phase and, consequently, its transformation into the spinel phase. However, its results were still very close to the results obtained for the other samples.

**Figure 9:**
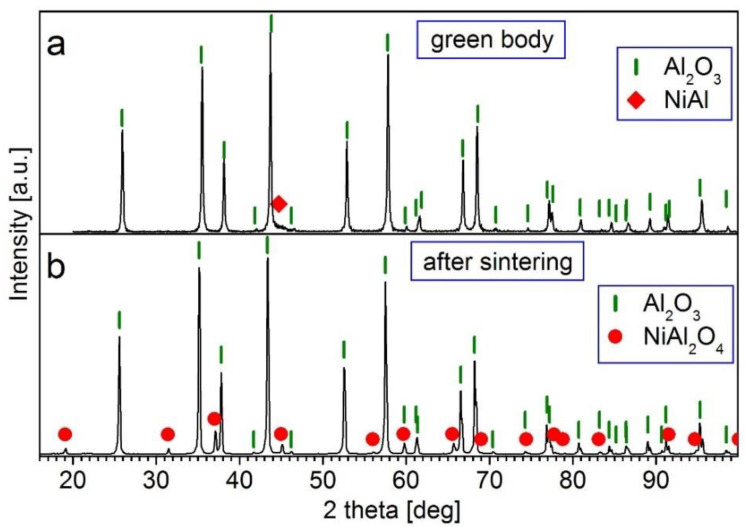
XRD patterns of the Al_2_O_3_ + 2.5 vol.% of (NiAl + 30 wt.% A_2_O_3_) sample: (**a**) green body; (**b**) after sintering at 1450 °C for 2 h.

**Figure 10:**
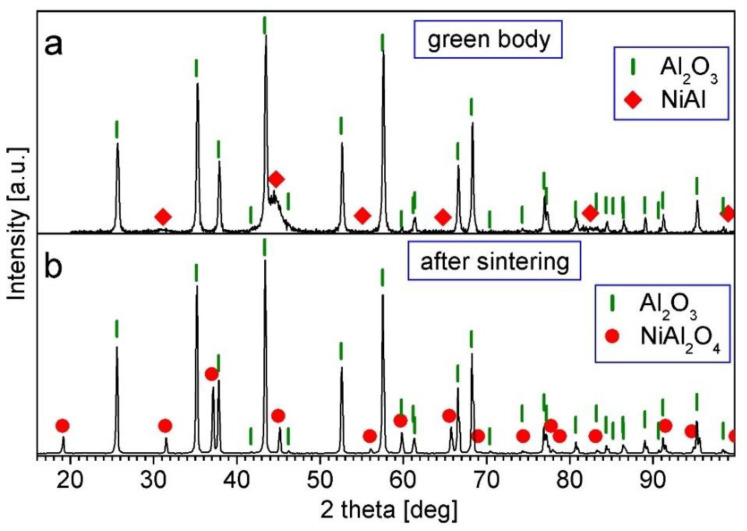
XRD patterns of the Al_2_O_3_ + 5 vol.% of (NiAl + 30 wt.% A_2_O_3_) sample: (**a**) green body; (**b**) after sintering at 1450 °C for 2 h.

Macroscopic observations of the composites revealed that the surface of the sintered samples had a pale turquoise color, indicating the formation of a new phase as spinel. Surface XRD analysis confirmed the NiAl_2_O_4_ spinel phase (Figure 9 and Figure 10).

Figure 9 and Figure 10 show the XRD patterns of the Series II and III samples before (green body) and after sintering in air at 1450 °C. In the patterns for the green bodies (Figure 9a and Figure 10a), diffraction peaks of the Al_2_O_3_ (ICDD 10-0137) and NiAl (ICDD 20-0019) phases were present. This shows that no reaction between the suspension components occurred during the mixing of the mechanically alloyed composite powder and Al_2_O_3_ powder in the PM400 planetary ball mill. On the contrary, in the XRD patterns of the sintered samples at the surface area (Figure 9b and Figure 10b), there were peaks of the Al_2_O_3_ (ICDD 10-0137) and NiAl_2_O_4_ (ICDD 10-0339) phases, whereas there were no peaks of the NiAl phase. This shows that a reaction between NiAl and a part of Al_2_O_3_ as well as the formation of a NiAl_2_O_4_ spinel occurred during sintering.

**Figure 11:**
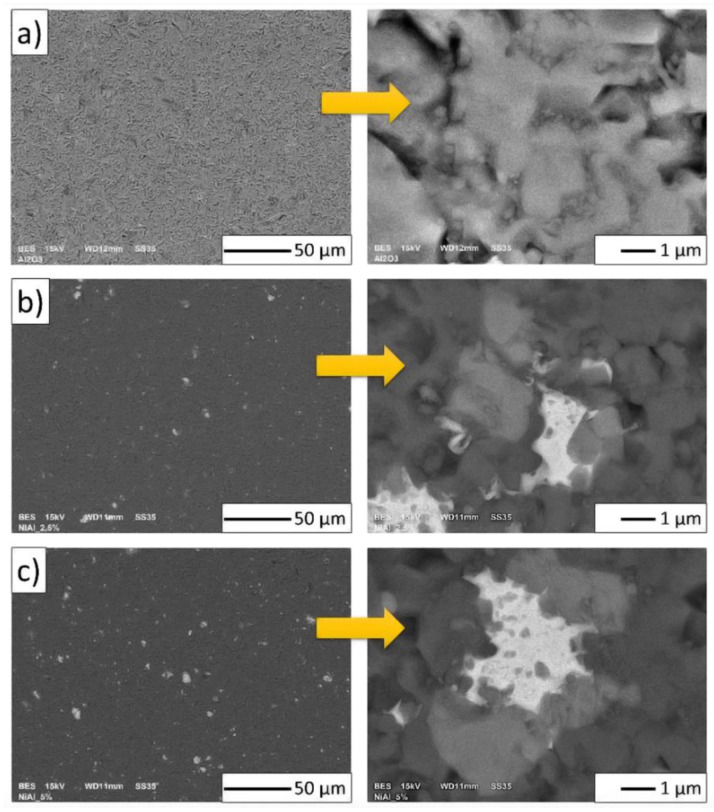
Microstructural images of the obtained samples: (**a**) Series I—100 vol.% of Al_2_O_3_; (**b**) Series II—Al_2_O_3_ + 2.5 vol.% of NiAl + 30 wt.% A_2_O_3_; (**c**) Series III—Al_2_O_3_ + 5 vol.% of NiAl + 30 wt.% A_2_O_3_.

In the central part of the composites, there was also the formation of NiAl_2_O_4_ spinel, which was expected. The SEM microstructural images of the obtained specimens are shown in Figure 11. The samples in Series I (pure Al_2_O_3_) contained only alumina (Figure 11a). For the composites (Figure 11b,c), the darkest areas probably corresponded to the Al_2_O_3_ phase, while the bright gray (almost white) and gray areas were the Ni and NiAl_2_O_4_ phases. EDX revealed the exact composition of the samples.

**Figure 12:**
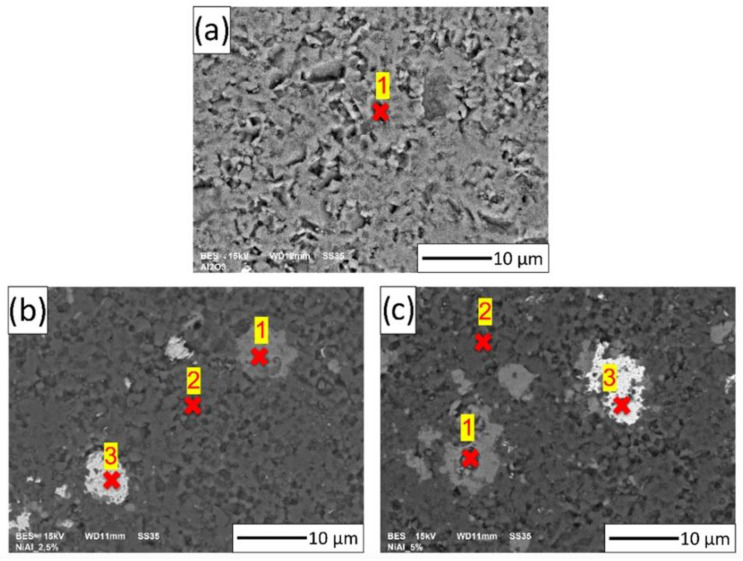
Analysis of the chemical composition for the selected points in the samples: (**a**) Series I—100 vol.% of Al_2_O_3_; (**b**) Series II—Al_2_O_3_ + 2.5 vol.% of NiAl + 30 wt.% A_2_O_3_; (**c**) Series III—Al_2_O_3_ + 5 vol.% of NiAl + 30 wt.% A_2_O_3_.

An energy-dispersive X-ray spectroscopy analysis was performed for selected points on the surfaces of all of the samples. EDX spectra were collected from one point for Series I and three different points for the composites (i.e., Series II and Series III). The points used to measure EDX are shown in Figure 12. The concentration measurements of the aluminum, nickel, and oxygen samples are presented in Table 3. The study showed that point 1 in the Series I sample contained 54.85 ± 0.05% aluminum and 45.15 ± 0.07% oxygen. For point 1 of the composite samples (i.e., Series II and Series III), a similar weight % ratio for all three elements was found, that is, for nickel, aluminum, and oxygen. These proportions of elements calculated in at.% (Series II Ni_14.0_Al_32.3_O_53.7_; Series III Ni_12.2_Al_31.8_O_56_) correlated with the spinel phase (Ni_14_Al_29_O_57_). For point 2 in the composite samples, the following values were recorded: 54.07 ± 0.14% aluminum and 45.93 ± 0.14% oxygen for Series II; 60.54 ± 0.16% aluminum and 39.46 ± 0.16% oxygen for Series III. These results revealed that point 2 corresponded to areas of the Al_2_O_3_ matrix in the case of the composites (i.e., Series II and III). However, for point 3 of Series II and III, an advantage for Ni was found. At this point, the content of the individual elements was as follows: Series II—92.92 ± 0.12% nickel, 3.19 ± 0.07% aluminum, and 3.89 ± 0.09% oxygen; Series III—96.19 ± 0.11% nickel, 1.57 ± 0.07% aluminum, and 2.24 ± 0.08% oxygen.

Furthermore, the EDX analysis showed that the element content at the investigated point 3 in the composites of Series II and Series III were noted as areas with high nickel content and low aluminum and oxygen concentrations. These results suggest that after air sintering, starting from the initial powder Al_2_O_3_ and compo-powder (NiAl-Al_2_O_3_), ceramic matrix composites with Ni and NiAl_2_O_4_ phases were obtained. However, it should be noted that in the area of Ni, fine particles of Al_2_O_3_ were visible (Figure 11).

Data in the literature indicate that sintering in an air atmosphere can lead to the formation of a spinel phase in the samples [32,33]. In the composites, the NiAl_2_O_4_ phase may have formed due to the reaction of Al or Ni with Al_2_O_3_ or oxygen from the gas phase according to the equation: Al_2_O_3_ + Ni + ½ O_2_ -> NiAl_2_O_4_ [34]. However, in the presented work with the initial powder, there was a NiAl compound. Some data show that oxygen dosing and annealing of the NiAl surface leads to oxygen diffusion into the bulk and nucleation of spinel [35]. Therefore, it can be concluded that during air sintering, a reaction among the components occurred in the case of the samples in Series II and Series III, which resulted in the formation of the NiAl_2_O_4_ spinel phase.

**Figure 13:**
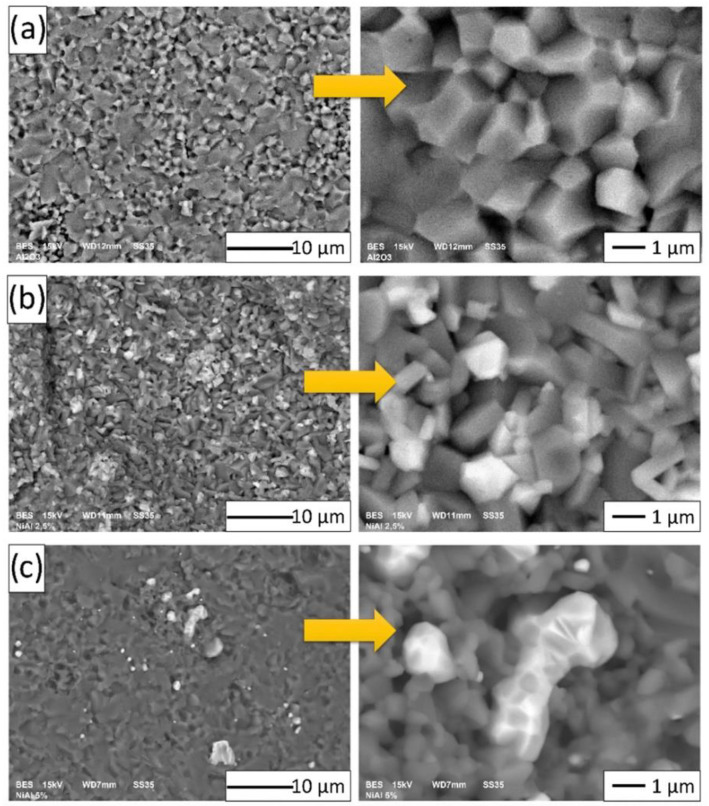
Scanning electron micrograph of the fractures in the fabricated samples: (**a**) Series I—100 vol.% of Al_2_O_3_; (**b**) Series II—Al_2_O_3_ + 2.5 vol.% of NiAl + 30 wt.% A_2_O_3_; (**c**) Series III—Al_2_O_3_ + 5 vol.% of NiAl + 30 wt.% A_2_O_3_.

Examination of the microstructures of the composites confirmed that the composites were well densified. The single voids visible in the microstructures (Figure 13) were due to the particles pulling out during the preparation of the samples. Generally, the Ni phase was distributed homogeneously in the ceramic matrix in both series of samples. 

The fracture microstructures of all of the prepared samples are presented in Figure 13. Fractures in the investigated materials were characterized by an intergranular brittle nature. From the SEM images, it may be concluded that intercrystalline fractures were observable along the grain boundaries. Therefore, the fracture surfaces were smooth and in the shape of polyhedrons corresponding to grain shapes. Based on observations, it was noted that there was a fracture at the grain boundary and not through the middle of the grain during sample breaking.

**Figure 14:**
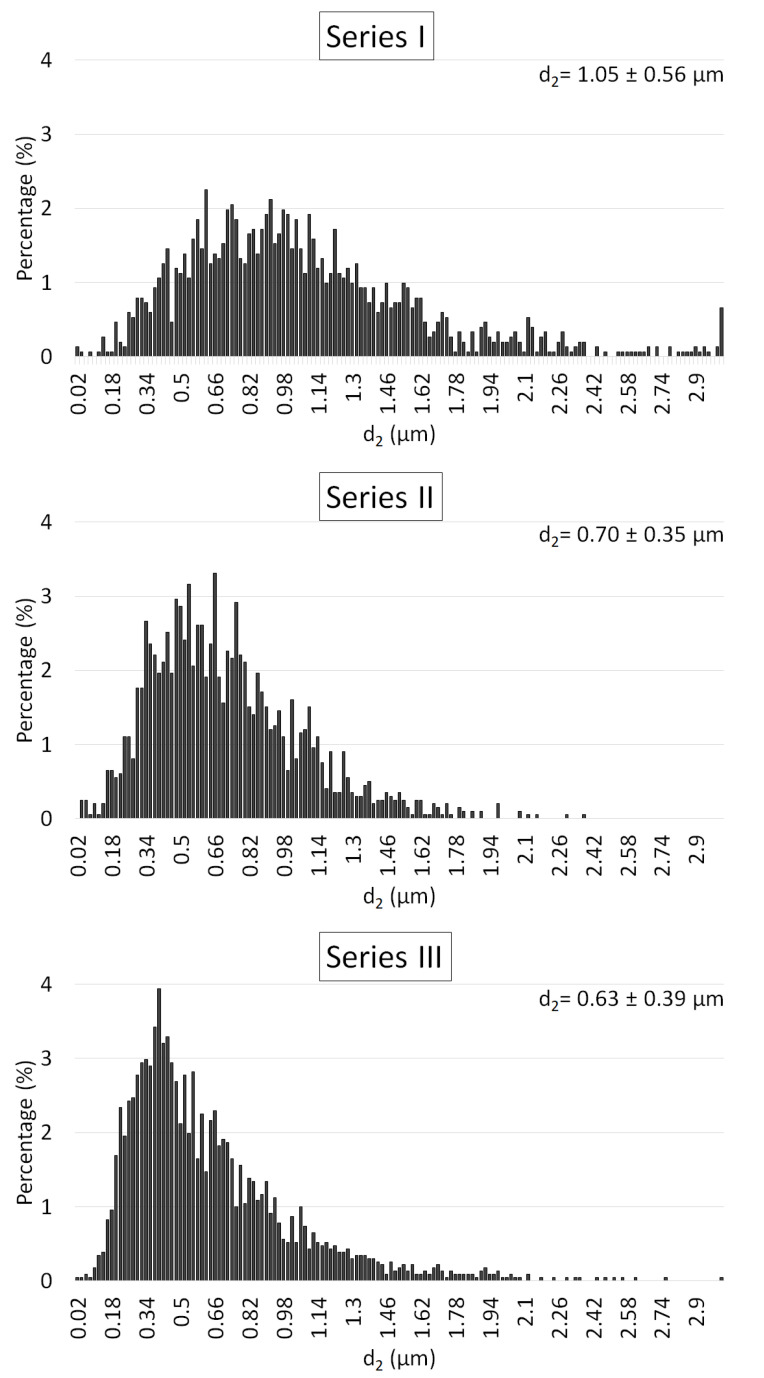
Histogram of the size distribution of Al_2_O_3_ grain in the samples produced.

In the next step, based on the obtained SEM microphoto (Figure 13), the influence of the compo-powder (NiAl-Al_2_O_3_) on the growth of Al_2_O_3_ grains in the composites was determined. Histograms obtained of the size distribution of Al_2_O_3_ in the samples produced are shown in Figure 14. These results demonstrated that the compo-powder slightly reduced the growth of Al_2_O_3_ grains. The measurements allowed us to note that the samples contained 100 vol.% of alumina characterized by an average grain size equal to 1.05 ± 0.56 µm. For Series I (100 vol.% Al_2_O_3_), sintering at 1450 °C caused the Al_2_O_3_ grain size to increase ten times over the starting powder (0.13 ± 0.07 µm). In the case of Series II (Al_2_O_3_ + 2.5 vol.% of compo-powder), the Al_2_O_3_ grain was characterized by an equal average size of 0.70 ± 0.35 µm. While in the samples from Series III containing 5 vol.% of compo-powder, Al_2_O_3_ was, on average, equal to the size 0.63 ± 0.39 µm. 

The parameters describing the Al_2_O_3_ shape factors in all of the samples are exhibited in Table 4. It can be found that similar shapes of Al_2_O_3_ grains characterized all of the samples. Similar values evidence this for the shape parameters, i.e., elongation, the curvature of the grain boundary, and convexity.

**Figure 15:**
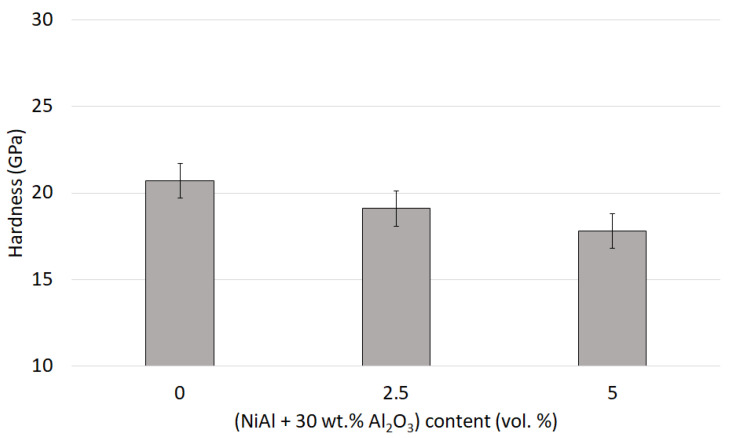
Hardness of the samples obtained.

In the next step, the focus was on testing the hardness and fracture toughness of the samples produced. The hardness values of the samples obtained are shown in Figure 15. 

The hardness examinations revealed a correlation between the content of using the initial compo-powder and the achieved hardness. Therefore, the reference samples in Series I (100 vol.% Al_2_O_3_) exhibited the highest hardness, with an average value of 20.70 ± 0.94 GPa. The use of the initial compo-powder caused, as expected, a decrease in the average hardness with the increase in the produced composites, and it was more significant for a higher content of the initial compo-powder. Therefore, the average hardness of the composites in Series II with a 2.5 vol.% (NiAl + 30 wt.% Al_2_O_3_) amounted to 19.10 ± 1.11 GPa, while that in Series III with a 5 vol.% (NiAl + 30 wt.% Al_2_O_3_) contribution to the structure was already at 17.80 ± 1.40 GPa. The observed decrease in the hardness for samples with a contribution of composite powder (NiAl-Al_2_O_3_) was the result of NiAl transformation into the spinel phase and, as a consequence, appearing in the microstructural metallic Ni phase. 

The hardness of the reference samples made of pure Al_2_O_3_ was consistent with the values obtained in the previous research and the data available in the literature. Previous research by our team showed that the hardness of the Al_2_O_3_ samples at the corresponding sintering temperature reached 21.00 ± 0.60 GPa [17]. Slightly lower values for pure Al_2_O_3_ were obtained in studies conducted by the Zmak team, where the Al_2_O_3_ samples produced using the slip casting method reached a hardness of 16.47 GPa [36]. In a study by Yang et al., where MgO-doped Al_2_O_3_ was used in the manufacturing process, the hardness obtained for the samples of Al_2_O_3_ sintered under less pressure ranged from 18 to 19 GPa [37]. Hence, although the use of the initial compo-powder (NiAl + Al_2_O_3_) in the composite structure deteriorated its hardness, the obtained values were still relatively high, approaching those observed in the literature for pure Al_2_O_3_. The hardness achieved for composites with the addition of 5 vol.% compo-powder was approximately 16 GPa, which agrees with the results obtained in [38]. This could be the result of the fine Al_2_O_3_ particles that remained from the initial composite powder and were located in the Ni areas of the final composite. The mechanism of hardening by the ceramic particles of the ductile metal phase here operated. 

**Figure 16:**
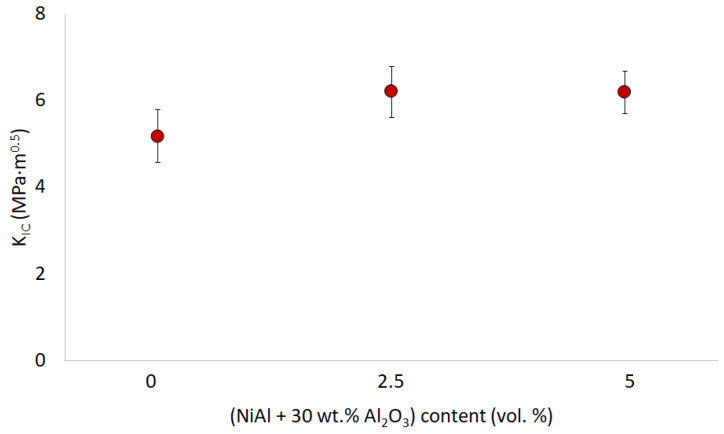
Fracture toughness of the samples obtained.

An analysis of the obtained results clearly show that using an initial compo-powder, even in small amounts, to fabricate the Al_2_O_3_ matrix composites improved the fracture toughness of the final material. While the K_IC_ value for the pure Al_2_O_3_ ceramics in this study was 5.18 ± 0.6 MPa·m^0.5^, in the samples using an initial compo-powder, it was 6.2 ± 0.59 MPa·m^0.5^ for the 2.5 vol.% of (NiAl + Al_2_O_3_) powder and 6.19 ± 0.49 MPa·m^0.5^ for the 5 vol.% of (NiAl + Al_2_O_3_) powder. The obtained fracture toughness values for the samples are shown in Figure 16. As can be seen, in both cases, these values exceeded those obtained for pure Al_2_O_3_. Simultaneously, these values were practically identical for the series with the addition of the initial compo-powder.

The K_IC_ results obtained for pure Al_2_O_3_ were slightly lower than in previous work carried out by our team, where the K_IC_ value for the Al_2_O_3_ samples sintered with PPS at 1400 °C, determined using the Anstis equation, reached 9.78 MPa·m^0.5^ [30]. However, the observed relationship correlated with results available for ceramic matrix composites in the literature. Although the final analyzed composites did not have a NiAl phase, the results can be compared to those of works on Al_2_O_3_-NiAl composites. Both in the work by Tuan et al., where the effect of NiAl additive on the structural and mechanical properties of the Al_2_O_3_ matrix composites was studied, and also in the research carried out by the team of Davies et al. [14,15,16], the addition of NiAl to the ceramic matrix in each of these works resulted in improved fracture toughness of the composite formed compared to pure Al_2_O_3_ ceramics.

## 4. Conclusions

This work presented the manufacturing of alumina matrix composites reinforced with Ni and NiAl_2_O_4_ phases. The initial composite powder (NiAl + 30 wt.% Al_2_O_3_) obtained by mechanical alloying was used to produce the composites. Slip casting was used as the sample formation method. Three series of samples were produced: Series I—100 vol.% of Al_2_O_3_; Series II—Al_2_O_3_ + 2.5 vol.% of (NiAl + 30 wt.% A_2_O_3_); Series III—Al_2_O_3_ + 5 vol.% of (NiAl + 30 wt.% A_2_O_3_).

The sinters were subjected to microstructural observations, X-ray diffraction analysis, and mechanical property measurements. The selected physical properties of the samples produced were determined. Additionally, image analysis was used to determine the effect of the metallic phase on the grain size of Al_2_O_3_.

The first research stage was prepared with a NiAl + 30 wt.% Al_2_O_3_ powder mixture by mechanical alloying, which was monitored based on XRD analysis. Based on the XRD results, it was found that after 5 h of mechanical alloying, peaks of a NiAl intermetallic phase appeared. It was noted that the intensity of the Ni and Al peaks gradually reduced with an increasing milling time and, finally, they vanished. The process of mechanical alloying of the powder lasted 18 h. Furthermore, the mean crystallite size of the NiAl phase in the final milling product, calculated using the Williamson–Hall method, was 10 nm.

The slip casting technique allowed for the samples characterized by a relative density close to 100% to be obtained. It was observed that within composites made from a more significant amount of initial compo-powder, the growth of Al_2_O_3_ grains in sinters decreased.

The EDX and XRD analyses showed that the sintering process of the samples in an air atmosphere caused the formation of a NiAl_2_O_4_ spinel phase in the composites. As a consequence, ceramic matrix composites with complex structures consisting of Ni and fine particles of Al_2_O_3_ that remained from the NiAl and newly formed NiAl_2_O_4_ phase were produced. The composites had high-quality sintered samples and favorable mechanical properties (i.e., high hardness and improved fracture toughness). This research opens a new path for the manufacture of ceramic–metal composites and will contribute to the development of modern composite materials.

## Figures and Tables

**Table 1:** Compositions of the prepared concentrated suspensions.

Component		Series I	Series II	Series III
Alumina oxide (Al_2_O_3_)	Vol.%	50	47.5	45
NiAl + 30 wt.% Al_2_O_3_	Vol.% with respect to the amount of ceramic	-	2.5	5
Diammonium hydrocitrate (DAC)	Wt.% with respect to the amount of ceramic or ceramic and metal powders	0.3		
Citric acid (CA)	Wt.% with respect to the amount of ceramic or ceramic and metal powders	0.1		
Water (H_2_O)	Vol.%	50		

**Table 2:** Selected physical properties of the samples.

Samples	Relative Density	Open Porosity	Soaking	Linear Shrinkage	Volume Shrinkage
	(%)	(%)	(%)	(%)	(%)
Series I—100 vol.% of Al_2_O_3_	99.66 ± 0.04	0.03 ± 0.01	<0.01	14.23 ± 0.04	35.97 ± 0.87
Series II—Al_2_O_3_ + 2.5 vol.% of NiAl + 30 wt.% A_2_O_3_	99.47 ± 0.34	0.05 ± 0.02	<0.01	14.77 ± 0.57	35.23 ± 0.61
Series III—Al_2_O_3_ + 5 vol.% of NiAl + 30 wt.% A_2_O_3_	99.05 ± 0.52	0.08 ± 0.02	<0.01	15.42 ± 0.75	34.91 ± 1.07

± Standard deviation.

**Table 3:** The weight content of the samples from different points.

**Series I—100 vol.% of Al_2_O_3_ (Figure 11a)**			
	Weight (%)		
	Ni	Al	O
Point 1	-	54.85 ± 0.05	45.15 ± 0.07
**Series II—Al_2_O_3_ + 2.5 vol.% of NiAl + 30 wt.% A_2_O_3_ (Figure 11b)**			
	Weight (%)		
	Ni	Al	O
Point 1	32.14 ± 0.27	34.18 ± 0.17	33.68 ±0.19
Point 2	-	54.07 ± 0.14	45.93 ± 0.14
Point 3	92.92 ± 0.12	3.19 ± 0.07	3.89 ± 0.09
**Series III—Al_2_O_3_ + 5 vol.% of NiAl + 30 wt.% A_2_O_3_ (Figure 11c)**			
	Weight (%)		
	Ni	Al	O
Point 1	28.92 ± 0.26	34.76 ± 0.17	36.32 ± 0.18
Point 2	-	60.54 ± 0.16	39.46 ± 0.16
Point 3	96.19 ± 0.11	1.57 ± 0.07	2.24 ± 0.08

± Standard deviation.

**Table 4:** Parameters describing shape factors of the Al_2_O_3_ grains in the samples.

Parameters Describing Shape Factors of Al_2_O_3_ Grains			
Type of Series	Elongation α = d_max_/d_2_	Convexity W = p/p_c_	Curvature of the Grain Boundary R = p/(π d_2_)
Series I—100 vol.% of Al_2_O_3_	1.38 ± 0.02	1.08 ± 0.01	1.25 ± 0.01
Series II—Al_2_O_3_ + 2.5 vol.% of NiAl + 30 wt.% A_2_O_3_	1.42 ± 0.03	1.07 ± 0.01	1.25 ± 0.01
Series III—Al_2_O_3_ + 5 vol.% of NiAl + 30 wt.% A_2_O_3_	1.42 ± 0.02	1.08 ± 0.01	1.27 ± 0.01

± Standard deviation. d_max_—maximum diameter of void projection (μm); d_2_—diameter of a circle of the same surface as the surface of the analyzed grain (μm); p_c_—Cauchy perimeter (μm); p—the perimeter of the void (μm) [25,26].

## Data Availability

Data sharing not applicable.

## References

[1] Yeomans J.A. (2008). Ductile particle ceramic matrix composites-scientific curiosities or engineering materials?. J. Eur. Ceram. Soc..

[2] Moya J.S., Esteban S.L., Pecharroman C. (2007). The challenge of ceramic/metal microcomposities and nanocomposities. Prog. Mater. Sci..

[3] Sun X., Yeomans Y. (1996). Optimization of a ductile particle-toughened ceramic. J. Am. Ceram. Soc..

[4] Ashby M.F., Brecht Y.J.M. (2003). Designing hybrid materials. Acta. Mater..

[5] Rodrigues-Suarez T., Bartolome J.F., Moya J.S. (2012). Mechanical and tribological properties of ceramic/metal composites: A revive of phenomena spanning from the nanometer to the micrometer length scale. J. Eur. Ceram. Soc..

[6] Xiang L., Wang F., Zhu J., Wang X. (2011). Mechanical properties and microstructure of Al_2_O_3_/TiAl in situ composites doped with Cr_2_O_3_. Mater. Sci. Eng. A.

[7] Tuan W.H., Pai Y.P. (1999). Mechanical properties of Al_2_O_3_-NiAl composites. J. Am. Ceram. Soc..

[8] Miracle D.B. (1993). Overview No. 104 The Physical and Mechanical Properties of NiAl. Acta Metall. Mater..

[9] Yan S.-R., Mehrizi M.Z., Foong L.K. (2020). Synthesis of NiAl-WC Composite by the Thermal Explosion of Elemental Powders. Ceram. Int..

[10] Zarezadeh Mehrizi M., Sedigh Mofrad S. (2021). Synthesis of NiAl/TiC–Al_2_O_3_ Composite by Mechanically Activated Combustion Synthesis. Ceram. Int..

[11] Krasnowski M., Gierlotka S., Kulik T. (2019). NiAl-B composites with nanocrystalline intermetallic matrix produced by mechanical alloying and consolidation. Adv. Powder Technol..

[12] Kaliński D., Chmielewski M., Pietrzak K., Choręgiewicz K. (2012). An Influence of Mechanical Mixing and Hot-Pressing on Properties of NiAl/Al_2_O_3_ Composite. Arch. Metall. Mater..

[13] Beyhaghi M., Kiani-Rashid A., Khaki J.V., Kashefi M., Jonsson S. (2019). Influences of Mechanical Activation and Heating Rate on Reaction Processes in Combustion Synthesis of NiAl-Al_2_O_3_ Composites. Powder Technol..

[14] Tuan W.H., Lin I.C., Pai Y.P., Chang S.T. (2000). Toughening Al_2_O_3_ with NiAl and NiAl(Fe) Particles. Br. Ceram. Trans..

[15] Tuan W.H., Chang S.T., Chou W.B., Pai Y.P. (2001). Effect of Milling Time on Mechanical Properties of Al_2_O_3_–NiAl Composites. Br. Ceram. Trans..

[16] Davies I.J., Pezzotti G., Bellosi A., Sciti D., Guicciardi S. (2002). Mechanical Behaviour of Nickel Aluminide Reinforced Alumina (Al_2_O_3_-NiAl) Composites. Adv. Compos. Lett..

[17] Konopka K., Zygmuntowicz J., Krasnowski M., Cymerman K., Wachowski M., Piotrkiewicz P. (2022). Pulse Plasma Sintering of NiAl- Al_2_O_3_ Composite Powder Produced by Mechanical Alloying with Contribution of Nanometric Al_2_O_3_ Powder. Materials.

[18] Michalski A., Jaroszewicz J., Rosinski M., Siemaszko D. (2006). NiAl-Al2O3 composites produced y puls plasma sintering with the participation of the SHS reaction. Intermetallics.

[19] Wachowski M., Zygmuntowicz J., Kosturek R., Konopka K., Kaszuwara W. (2021). Manufacturing of Al_2_O_3_/Ni/Ti Composites Enhanced by Intermetallic Phases. Materials.

[20] Gadow R., Kern F., Sarin V.K. (2014). 2.06—Advanced Manufacturing of Hard Ceramics. Comprehensive Hard Materials.

[21] Yüzbasi N.S., Graule T., Pomeroy M. (2021). Colloid Casting Processes: Slip Casting, Centrifugal Casting, and Gel Casting. Encyclopedia of Materials: Technical Ceramics and Glasses.

[22] (2018). Standard Terminology Relating to Catalysts and Catalysis.

[23] (1993). Advanced Technical Ceramics–Determination of Density and Porosity.

[24] Suryanarajana C., Grant Norton M. (1998). X-ray Diffraction. A Practical Approach.

[25] Michalski J., Wejrzanowski T., Pielaszek R., Konopka K., Łojkowski W., Kurzydłowski K.J. (2005). Application of image analysis for characterization of powders. Mater. Sci. Pol..

[26] Wejrzanowski T., Spychalski W., Rożniatowski K., Kurzydłowski K. (2008). Image Based Analysis of Complex Microstructures of Engineering Materials. Int. J. Appl. Math. Comput. Sci..

[27] Anstis G., Chantikul P., Lawn B., Marshall D. (1981). A Critical Evaluation of Indentation Techniques for Measuring Fracture Toughness: I, Direct Crack Measurements. J. Am. Ceram. Soc..

[28] Zygmuntowicz J., Piotrkiewicz P., Gizowska M., Tomaszewska J., Suchecki P., Wachowski M., Torzewski J., Żurowski R. (2022). The Potential of Al_2_O_3_–ZrO_2_-Based Composites, Formed via CSC Method, in Linear Infrastructure Applications Based on Their Mechanical, Thermal and Environmental Performance. Metall. Mater Trans. A.

[29] Krasnowski M., Gierlotka S., Ciołek S., Kulik T. (2019). Nanocrystalline NiAl intermetallic alloy with high hardness produced by mechanical alloying and hot-pressing consolidation. Adv. Powder Technol..

[30] Konopka K., Krasnowski M., Zygmuntowicz J., Cymerman K., Wachowski M., Piotrkiewicz P. (2021). Characterization of Al_2_O_3_ Samples and NiAl–Al_2_O_3_ Composite Consolidated by Pulse Plasma Sintering. Materials.

[31] Krasnowski M. (2017). Phase Transformations during Mechanical Alloying and Subsequent Heating of FeAlB Powders. J. Alloys Compd..

[32] Lieberthal M., Kaplan W.D. (2001). Processing and Properties of Al_2_O_3_ Nanocomposites Reinforced with Sub-Micron Ni and NiAl_2_O_4_. Mater. Sci. Eng. A.

[33] Tuan W.H., Lin M.C. (1996). Reaction Sintering of Al_2_O_3_/NiAl_2_O_4_ Composites. J. Mater Sci. Lett..

[34] Zygmuntowicz J., Wiecińska P., Miazga A., Konopka K. (2016). Characterization of Composites Containing NiAl_2_O_4_ Spinel Phase from Al_2_O_3_/NiO and Al_2_O_3_/Ni Systems. J. Therm. Anal. Calorim..

[35] Loginova E., Cosandey F., Madey T.E. (2007). Nanoscopic Nickel Aluminate Spinel (NiAl_2_O_4_) Formation during NiAl(111) Oxidation. Surf. Sci..

[36] Žmak I., Ćorić D., Mandić V., Ćurković L. (2020). Hardness and Indentation Fracture Toughness of Slip Cast Alumina and Alumina-Zirconia Ceramics. Materials.

[37] Yang S., Yang S., Zhu Y., Fan L., Zhang M. (2022). Flash Sintering of Dense Alumina Ceramic Discs with High Hardness. J. Eur. Ceram. Soc..

[38] Tuan W.H., Chou W.B., You H.C., Chang S.T. (1998). The Effects of Microstructure on the Mechanical Properties of Al_2_O_3_-NiAl Composites. Mater. Chem. Phys..

